# The Role of Cardiolipin as a Scaffold Mitochondrial Phospholipid in Autophagosome Formation: In Vitro Evidence

**DOI:** 10.3390/biom11020222

**Published:** 2021-02-05

**Authors:** Valeria Manganelli, Antonella Capozzi, Serena Recalchi, Gloria Riitano, Vincenzo Mattei, Agostina Longo, Roberta Misasi, Tina Garofalo, Maurizio Sorice

**Affiliations:** 1 Department of Experimental Medicine, Sapienza University, 00161 Rome, Italy; valeria.manganelli@uniroma1.it (V.M.); antonella.capozzi@uniroma1.it (A.C.); serena.recalchi@uniroma1.it (S.R.); gloria.riitano@uniroma1.it (G.R.); agostina.longo@uniroma1.it (A.L.); roberta.misasi@uniroma1.it (R.M.); tina.garofalo@uniroma1.it (T.G.); 2 Laboratory of Experimental Medicine and Environmental Pathology, 02100 Rieti, Italy; vincenzo.mattei@uniroma1.it

**Keywords:** cardiolipin, MAMs, autophagosome, mitochondria

## Abstract

Cardiolipin (CL) is a hallmark phospholipid localized within the inner mitochondrial membrane. Upon several mitochondrial stress conditions, CL is translocated to specialized platforms, where it may play a role in signaling events to promote mitophagy and apoptosis. Recent studies characterized the molecular composition of MAM-associated lipid microdomains and their implications in regulating the autophagic process. In this study we analyzed the presence of CL within MAMs following autophagic stimulus and the possible implication of raft-like microdomains enriched in CL as a signaling platform in autophagosome formation. Human 2FTGH fibroblasts and SKNB-E-2 cells were stimulated under nutrient deprivation with HBSS. MAM fraction was obtained by an ultracentrifugation procedure and analyzed by HPTLC immunostaining. CL interactions with mitofusin2 (MFN2), calnexin (CANX) and AMBRA1 were analyzed by scanning confocal microscopy and coimmunoprecipitation. The analysis revealed that CL accumulates in MAMs fractions following autophagic stimulus, where it interacts with MFN2 and CANX. It associates with AMBRA1, which in turn interacts with BECN1 and WIPI1. This study demonstrates that CL is present in MAM fractions following autophagy triggering and interacts with the multimolecular complex (AMBRA1/BECN1/WIPI1) involved in autophagosome formation. It may have both structural and functional implications in the pathophysiology of neurodegenerative disease(s).

## 1. Introduction

Cardiolipin (CL) is a hallmark lipid of mitochondria almost exclusively found within the inner mitochondrial membrane (IMM) [1], where it is synthesized and constitutes 

About 20% of the total lipid composition [2]. CL is required for optimal mitochondrial function and is known to give essential structural and functional support to several proteins involved in mitochondrial processes, including respiration and energy conversion [3,4,5]. Although the distribution of CL is mostly confined to the IMM, it also distributes in the outer mitochondrial membrane (OMM), specifically in the contact sites formed between the inner and outer membranes, where its content is even higher (about 27% of total phospholipids) [6]. These contact sites represent a relevant portion of mitochondrial membrane, since about 115 such sites were calculated in one isolated mitochondrion (ranging from 78 to 270 depending on cell type) [7]. The peculiar biochemical properties of CL have encouraged the hypothesis that membrane sites enriched with this lipid could function as binding sites for specific protein complexes, in fact two negative charges of CL allow the recruitment of specific protein interaction partners. In addition, it is known that CL can shift from IMM to OMM following mitochondrial stress conditions [8,9]. Thus, CL, which is translocated from the IMM and exposed on the OMM upon several mitochondrial stress conditions, may readily serve as a binding site for cytosolic proteins, thus establishing a hub for many cellular signaling events to promote mitophagy and apoptosis [10]. In fact, CL can also serve as a recognition signal for dysfunctional mitochondria by generating a binding platform for essential players of autophagic machinery, including Beclin 1 and LC3 [8], and of apoptotic signaling pathways such as tBid, Bax and caspase-8 [11,12,13]. 

Our previous works indicated that CL is a constituent of specialized platforms on mitochondrial membrane structure called raft-like microdomains [14]. These dynamic structures are enriched in gangliosides (GD3, GM3), but show a relatively low content of phospholipids and, unlike plasma membranes lipid raft, of cholesterol [15]. In these microdomains, some molecules, including the voltage-dependent anion channel-1 (VDAC-1) and the fission protein hFis1, are enriched [16], whereas Bcl-2 family proteins (truncated Bid, t-Bid, and Bax) are recruited following CD95/Fas triggering. These microdomains represent preferential sites on the mitochondrial membrane where some key reactions can be catalyzed, thus contributing to cell apoptosis [17,18,19]. CL within ‘‘raft-like” microdomains could anchor caspase-8 at contact sites between inner and outer membranes, facilitating its self-activation, Bid’s cleavage and cytochrome c release [20]. Recently, lipid “raft-like” microdomains have been identified as further actors of the autophagic process. 

Autophagy is an evolutionarily conserved process, in which intracellular membrane structures sequester proteins and organelles for lysosomal degradation [21]. Autophagy plays an important role in many biological processes related to cell survival and death [22,23,24]. Under oxidative stress and starvation conditions the production of reactive oxygen species (ROS) increases, leading to autophagy activation [25,26]. Autophagy is a lysosome-mediated, multistep self-degradation process during which components are degraded to supply energy, it is distinct from other degradative pathways such as proteasomal degradation [24,27]. Once specific multimeric protein complexes have been activated, an isolation membrane sequesters a small portion of the cytoplasm, including cytosolic materials and organelles, to form the autophagosome, spherical double-membrane vesicles generated from several membrane sources, including the endoplasmic reticulum (ER) and mitochondria [28]. Autophagosomes fuse with lysosomes to yield autolysosomes, which degrade internalized materials. Generation of the phagophore, the morphological step that precedes the autophagosome formation, requires the BECN1/Beclin 1-class III phos-phatidylinositol (PtdIns) 3-kinase (PtdIns3K) complex, as well as generation and insertion of LC3 (microtubule-associated protein 1 light chain 3)-II into the autophagosomal membrane. This is a consistent key step in autophagosomal formation in which sphingolipids are known to contribute [16,29,30,31]. 

Emerging evidence indicates that membrane isolation is tightly coordinated by particular subdomains, named mitochondria-associated membranes (MAMs), which represent 5–20% of the mitochondrial surface in close contact with the ER in resting conditions. This percentage dynamically increases following various cell stresses [32,33,34,35]. The presence of lipid domains in the MAM vesicles has been reported by several authors [36,37]. Moreover, an increasing number of large-scale proteomics studies characterized the molecular composition of MAM-associated lipid microdomains [38] and their implications in regulating and influencing a variety of cellular activities [39], including the autophagic process [37,40,41]. 

In this study we analyzed the presence of CL within MAMs following autophagic stimulus and the possible implication of raft-like microdomains enriched in CL as a signaling platform in autophagosome formation.

## 2. Materials and Methods 

### 2.1. Cells and Autophagy Induction

Human 2FTGH (2F) fibroblasts (provided by ECACC, 12021508) and Human Neuroblastoma SK-N-BE2 cell lined (ATCC Cat# CRL-2271) were grown in Dulbecco’s modified Eagle’s medium (DMEM; Sigma-Aldrich, Milan, Italy, D5796), containing 10% fetal calf serum (FCS) plus 100 units/mL penicillin, 10 mg/mL streptomycin, at 37 °C in a humified CO_2_ atmosphere. Autophagy was induced by nutrient deprivation through the use of the HBSS medium (Sigma-Aldrich, H9269). The optimal incubation time of the cells in HBSS was determined in preliminary experiments and was for 1 h at 37 °C. After treatment, cells were collected and prepared for the experimental procedures described below. 

### 2.2. Isolation of MAMs

The isolation of the high-purity MAM and Mitochondria fractions were obtained with the method described by Wieckowski et al. [42]. Briefly, crude mitochondria, obtained from human skin fibroblasts, untreated or treated with HBSS for 1 h at 37 °C, were subjected to differential centrifugation steps to get a partly pure “crude” mitochondrial fraction and a supernatant fraction enriched in ER and cytosol. Further purification was achieved by Percoll gradient ultracentrifugation. Two ml of ice-cold mitochondria resuspension buffer (MRB), containing 250 mM mannitol (Sigma-Aldrich, M9546), 5 mM HEPES (Sigma, H-0887), pH 7.4, 0.5 mM EGTA were used to resuspend the crude mitochondrial pellets, which were then layered over 8 mL of Percoll medium (225 mM Mannitol, 25 mM HEPES, pH 7.4, 1 mM EGTA and 30% Percoll (vol: vol; Sigma-Aldrich, P164)). Afterwards, the tube was gently filled up with 3.5 mL of MRB solution and centrifuged at 95,000× *g* for 30 min at 4 °C in an SW 41 rotor (Beckman Institute, Brea, CA, USA). After centrifugation, 2 bands were distinguished, the densest and closest to the bottom containing the isolated mitochondria and the diffuse white band above constituted the MAM fraction. The fractions were collected and centrifuged at 6300× *g* for 10 min at 4 °C. MAM supernatant was subjected to a further centrifugation at 100,000× *g* for 1 h at 4 °C in a 70-Ti rotor (Beckman). After evaluation of the protein concentration by Bradford Dye Reagent assay (Bio-Rad, Hercules, CA, USA, 500006), MAMs and pure mitochondria fractions were analyzed by Western blot analysis using anti-CANX MAb (Abcam, Cambridge, UK, 6F12BE10), anti-VDAC1 MAb (Santa Cruz Biotechnology, Inc., Heidelberg, Germany, sc-390996) and anti-Cyt c PAb (Abcam, ab90529).

### 2.3. CL Extraction and Analysis by HPTLC-Immunostaining

The MAM fractions and pure mitochondria were subjected to phospholipid extraction for CL analysis. Briefly, both samples, MAM and pure mitochondria, were treated with 1 mL of methanol and subsequently with 2 mL of chloroform and mixed vigorously for 5 min. Then, after the addition of 0.5 mL of 0.15 M NaCl, the organic phase was separated by centrifugation, dried under nitrogen, dissolved in chloroform and re-centrifuged to remove any insoluble material. The extract thus obtained was analyzed by high performance thin layer chromatography (HPTLC) immunostaining, performed as described before [43]. Phospholipids were separated using HPTLC with aluminum-backed silica gel 60 (20 × 20) plates (Merck, Darmstadt, Germany). As liquid phase, an eluent system of chloroform/methanol/acetic acid/water (100:75:7:4, *v*/*v*/*v*/*v*) was used. The dried chromatograms were soaked for 90 s in a 0.5% (*w*:*v*) solution of poly(isobutyl methacrylate) beads (Polysciences, Eppelheim, Germany) dissolved in hexane. After air-drying for 5 min, silica plates were incubated at room temperature for 1 h with 1% bovine serum albumin (BSA) in phosphate-buffered saline (PBS) as blocking buffer. After 3 gentle shaking washes for 10 min in PBS containing 0.1% Tween 20 (PBS-T), the plates were incubated for 1 h at room temperature with a human anti-Cardiolipin antibody (MyBioSource, San Diego, CA, USA, MBS613746; final dilution 1:500) and then with HRP-conjugated anti-human IgG (Sigma-Aldrich). Immunoreactivity was assessed by chemiluminescence reaction using the ECL Western detection system (Amersham Biosciences, Buckinghmashire, UK). 

### 2.4. Western Blot Analysis

Cells, untreated or treated with HBSS for 1 h at 37 °C, were lysed in lysis buffer, containing 1% Triton X-100 (Bio-Rad, 1610407), 10 mM Tris-HCl, pH 7.5, 150 mM NaCl, 5 mM EDTA, 1 mM Na_3_VO_4_ (Sigma Aldrich, 450243), 75 U of aprotinin (Sigma-Aldrich, A1153) for 20 min at 4 °C. Centrifugation for 5 min at 1300× *g* removed nuclei and large cell debris from the lysate. The protein concentration of the sample was evaluated by the Bradford Dye Reagent assay (Bio-Rad, 500-0006), then the 15% sodium-dodecyl sulfate polyacrylamide gel electrophoresis (SDS-PAGE) was performed, with subsequent electrophoretic transfer of proteins on polyvinylidene difluoride (PVDF) membranes (Bio-Rad, 162-0177). As a blocking solution we used 5% fat-free milk powder in TBS (Bio-Rad, 1706435), containing 0.05% Tween 20 (Bio-Rad, 1706531), for 1 h at room temperature. Subsequently, the membranes were probed with rabbit anti-LC3 PAb (Novus Biologicals, Centennial, CO, USA, NB100-2331) or anti-p62/SQSTM1 PAb (Abcam, ab91526) and horseradish peroxidase (HRP) conjugated anti-rabbit IgG (Sigma-Aldrich, A1949) as a secondary antibody. Immunoreactivity was assessed by chemiluminescence reaction, using the ECL Western detection system (Amersham, RPN2106). Densitometric scanning analysis was performed with Mac OS X (Apple Computer International, Cupertino, CA, USA), using NIH ImageJ 1.62 software or by Image Lab software from Bio-Rad version 6.0.1.

### 2.5. Immunoprecipitation Experiments

Cells, untreated or treated with HBSS for 1 h at 37 °C, were lysed in lysis buffer (10 mM Tris-HCl, pH 8.0, 150 mM NaCl, 1% Nonidet P-40 (Sigma-Aldrich, 213277), 1 mM phenylmethylsulfonyl fluoride (Sigma-Aldrich, P7626), 10 mg/mL leupeptin (Sigma-Aldrich, L2884). In order to preclear nonspecific binding, cell-free lysates were mixed with protein G-acrylic beads (Sigma-Aldrich, P3296) and stirred by a rotary shaker for 2 h at 4°C. After centrifugation (500× *g* for 1 min) to remove beads, the supernatant fraction was incubated with rabbit anti-CANX PAb (Sigma-Aldrich, C4731), or rabbit anti-AMBRA1 PAb (Covalab, Bron, France, 0224), or rabbit anti-MFN2 PAb (Abcam) at 4 °C for 1 h. The antigen/Ab complexes were immunoprecipitated by addition of protein G-acrylic beads for 1 h at 4 °C and subsequent centrifugation for 1 min at 500× *g* at 4 °C. As a negative control, immunoprecipitation was performed with an irrelevant rabbit IgG (Sigma-Aldrich, 15006). The immunoprecipitates were split into 2 aliquots and washed once in PBS. The first aliquot was subjected to phospholipid extraction and analyzed by HPTLC immunostaining analysis for CL detection; the second one was checked by Western blot analysis using anti-MFN2 MAb (Cell Signaling Technology, D1E9 11925S), anti-CANX MAb (Abcam, ab31290), anti AMBRA1 MAb (Santa Cruz Biotechnology, sc-398204), anti-BECN1 PAb (Santa Cruz Biotechnology, sc-11427) and anti-WIPI1 PAb (Bioss, Woburn, MA, USA, ABIN750418).

### 2.6. Analysis of CL Localization by Scanning Confocal Microscopy

Human 2FTGH (2F) fibroblasts cells, untreated or treated with HBSS for 1 h at 37 °C were fixed with 4% paraformaldehyde for 30 min and then permeabilized with 0.5% Triton X-100 in PBS for 5 min at room temperature. Cells were stained with human anti-CL (MyBioSource) and rabbit anti-MFN2 (Abcam) or rabbit anti-AMBRA1 (Covolab) for 1 h at 4 °C, followed by three washes in PBS and addition (1 h at 4 °C) of Texas Red-conjugated anti-rabbit IgG (Thermo Fisher Scientific, Rome, Italy, T-2767) and FITC-conjugated anti-human IgG (Sigma-Aldrich, F9512). After incubation, the cells were washed three times with PBS and then resuspended in 0.1 M Tris-HCl, pH 9.2, containing 60% glycerol (*v*:*v*). The images were acquired with Olympus FV1000 spectral confocal laser scanning microscope.

### 2.7. Statistical Analysis

All the statistical procedures were performed by GraphPad Prism software Inc. (San Diego, CA, USA). All data reported in this paper were verified in at least 3 different experiments performed in duplicate and reported as mean ± standard deviation (SD). *p* values for all graphs were generated using Student’s *t*-test as indicated in the figure legends; * *p* ≤ 0.05, ** *p* ≤ 0.005 *** *p* ≤ 0.001, **** *p* ≤ 0.0001. 

## 3. Results

### 3.1. Cardiolipin Accumulates in MAMs Fractions Following Autophagic Stimulus 

**Figure 1:**
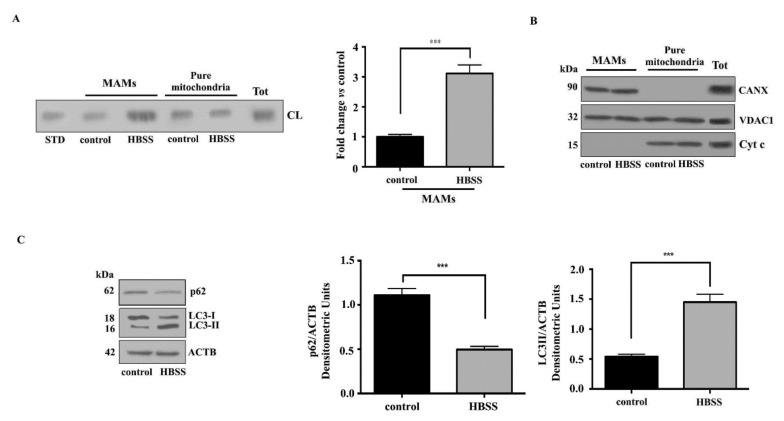
Cardiolipin translocates to MAM subdomains during autophagy. (**A**) Isolated MAMs and pure mitochondria fractions obtained from human 2FTGH (2F) fibroblasts cells, untreated or treated with HBSS for 1 h at 37 °C were subjected to phospholipid extraction. All fractions were run on HPLC plates and analyzed by HPTLC immunostaining for the presence of CL using anti-CL as described in Materials and Methods. A positive control was obtained using pure standard CL (STD, Sigma-Aldrich). (**B**) The purity of MAMs and pure mitochondria fractions was also tested by Western blotting for the presence of specific markers: CANX for MAMs or VDAC1 for mitochondria and MAMs, Cytc for mitochondria. (**C**) Autophagy was checked by Western blot analysis, using rabbit anti-LC3 PAb or rabbit anti-p62/SQSTM1 pAb. Loading control was evaluated using anti-ACTB Mab. A representative experiment among 3 is shown. Bar graph on the right shows densitometric analysis. Results represent the mean ± SD from 3 independent experiments. *** *p ≤* 0.001, HBSS vs. control cells.

MAMs have been proposed to contain membrane microdomains that are enriched in cholesterol and glycosphingolipids [36,37]. We previously reported that the presence of lipid rafts in the MAMs could be pivotal in the mitochondria–ER crosstalk leading to autophagosome formation [37]. Since CL may be present in raft-like microdomains at contact sites between inner and outer mitochondrial membranes, we first tested the presence of CL in MAM fractions. With this aim, crude mitochondrial fractions were sub-fractionated to obtain both MAMs and pure mitochondria fractions from Human 2FTGH (2F) fibroblasts following incubation for 1 h with HBSS, a physiological inducer of autophagy. To verify whether CL is present in isolated MAMs fractions, we analyzed its distribution in MAMs and pure mitochondria fractions under starvation conditions. Both fractions were first subjected to phospholipid extraction and then analyzed by HPTLC-Immunostaining for the presence of CL. The analysis revealed an anti-CL antibody positive band both in control and in treated cells, suggesting that CL was constitutively present in isolated MAMs fractions, although it is more evident in pure mitochondria preparation (Figure 1A, left panel). After autophagy induction, CL content in MAM fraction was significantly increased, as confirmed by densitometric analysis (Figure 1A, right panel). The purity of MAMs preparations was assessed by checking the presence of specific markers: CANX (calnexin) for MAMs, VDAC1 (voltage dependent anion channel 1) for mitochondria and MAMs, Cyt c (Cytochrome c) for mitochondria (Figure 1B). Autophagy induction was checked in control and HBSS-treated cells by Western blot analysis, using anti-LC3 (microtubule associated protein 1 light chain 3) or anti-p62/SQSTM1 (sequestosome 1) antibodies (Figure 1C). Western blot analysis revealed an increase of LC3-II after cell starvation, together with a significant decreased of p62, which was also confirmed by densitometric analysis.

### 3.2. Interaction of Cardiolipin with MFN2 and Calnexin within MAMs

**Figure 2:**
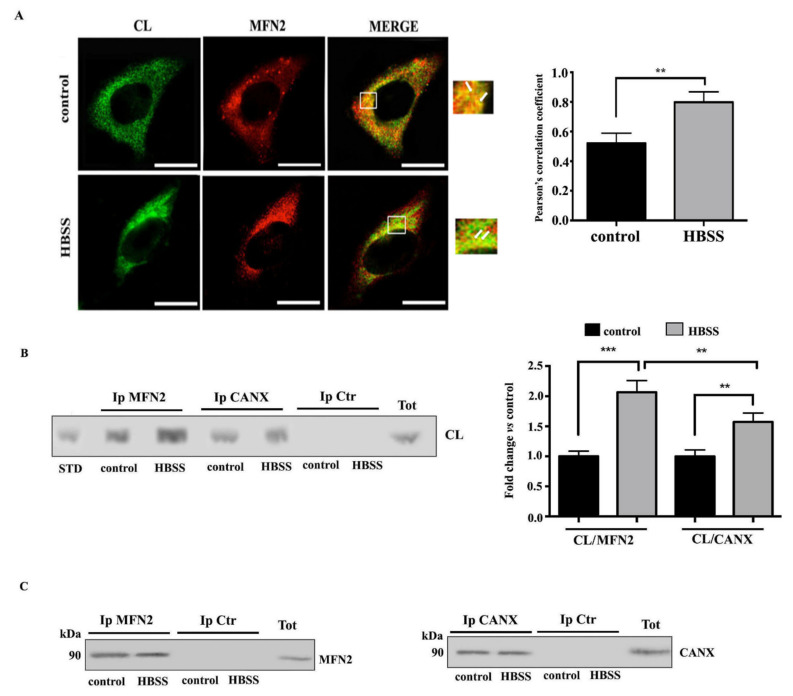
CL interaction with MFN2 and CANX during autophagy induction in fibroblast cells. *(***A**) Human 2FTGH (2F) fibroblasts cells, untreated or treated with HBSS for 1 h at 37 °C were fixed with 4% paraformaldehyde for 30 min and then permeabilized with 0.5% Triton X-100 in PBS for 5 min at room temperature. Cells were stained with rabbit anti-MFN2 and human anti-CL for 1 h at 4 °C, followed by addition of Texas Red-conjugated anti-rabbit IgG and FITC-conjugated anti-human IgG (Sigma-Aldrich). The images were acquired with Olympus FV1000 spectral confocal laser scanning microscope. The Image J Just Another Colocalization Plugin (JACoP) was applied for quantitative colocalization analyses. Pearson’s correlation coefficient was calculated. A minimum of 30 cells/sample was analyzed and the statistical analysis was performed using Student’s *t*-test, ** *p* ≤ 0.01. Scale bar, 5 μm. *(***B**) Human 2FTGH (2F) fibroblasts cells, untreated or treated with HBSS for 1 h, were lysed in lysis buffer, followed by immunoprecipitation with rabbit anti-MFN2 PAb or rabbit anti-CANX PAb. A rabbit IgG isotypic control (IpCtr) was employed. The immunoprecipitates were subjected to phospholipid extraction and analyzed by HPTLC immunostaining for the presence of CL. A positive control was obtained using pure standard CL (STD, Sigma-Aldrich). (**C**) The immunoprecipitates were checked by Western blot analysis, using mouse anti-MFN2 MAb or mouse anti-CANX MAb. A representative experiment among 3 is shown. Bar graph on the right shows densitometric analysis. Results represent the mean ± SD from 3 independent experiments. ** *p* ≤ 0.01, and *** *p* ≤ 0.001, HBSS vs control cells.

Hence, since CL is constitutively present within MAMs during the autophagic process, we analyzed whether CL may interact with key molecules involved in the early events of autophagic process within MAMs. It is known that MFN2 builds a bridge between ER and mitochondria, supplying autophagosomes membranes and regulating the autophagic flux. In the light of this, we investigated a possible molecular association of CL with MFN2 during autophagy by scanning confocal microscopy. As expected, laser confocal microscopy showed an association of CL with MFN2 in control cells. In fact, the overlays of CL (green) and MFN2 (red) showed extensive yellow areas indicative of strong colocalization (Figure 2A). These areas of colocalization were significantly increased in cells stimulated with HBSS for 1 h, where they formed characteristic “dots”, reminiscent of a typical increase of MAMs following HBSS triggering. This result was also confirmed by coimmunoprecipitation experiments. Thus, cell-free lysates from HBSS-treated and untreated cells were immunoprecipitated with anti-MFN2 PAb, followed by protein G-acrylic beads. Phospholipids were extracted from MFN2 immunoprecipitates and analyzed by HPTLC immunostaining. The analysis revealed an anti-CL antibody positive band, comigrating with standard CL in untreated cells, which became more evident after triggering through HBSS, as revealed by densitometric analysis. In addition, we also evaluated the possible interaction between CL and CANX, a prototypical Ca2+-binding ER palmitoylated chaperone protein enriched in the MAMs. These results revealed a weak interaction between CL and CANX both in unstimulated and in HBSS-treated cells. In contrast, no bands were revealed in the immunoprecipitates from control cells or from cells immunoprecipitated with IgG with irrelevant specificity, obtained under the same experimental conditions (Figure 2B). The immunoprecipitation was verified by Western blot (Figure 2C). 

### 3.3. Cardiolipin Interacts with AMBRA1/BECN1/WIPI1 Complex during Autophagosome Formation

**Figure 3:**
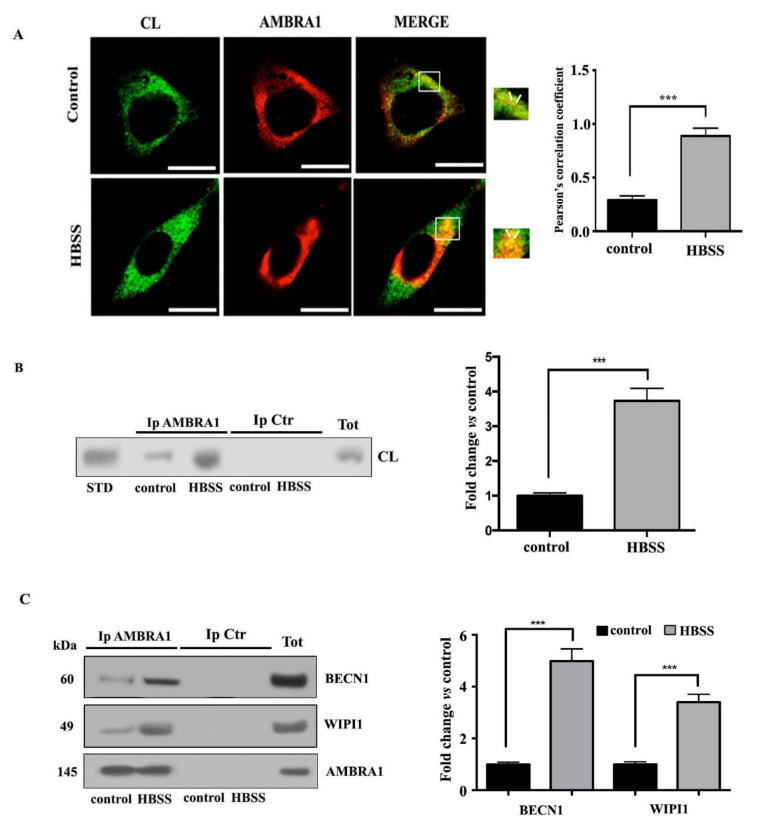
Cardiolipin/AMBRA1 association during autophagy induction in fibroblast cells. (**A**) Human 2FTGH (2F) fibroblasts untreated or treated with HBSS for 1 h were fixed with 4% paraformaldehyde in PBS for 30 min at room temperature and then permeabilized by 0.5% Triton X-100 in PBS for 5 min at room temperature; then cells were incubated with human anti-CL and rabbit anti-AMBRA1 PAb for 1 h. After washing, cells were incubated with Texas Red-conjugated anti-rabbit IgG or with FITC-conjugated anti-human IgG. The images were acquired with Olympus FV1000 spectral confocal laser scanning microscope. Pearson’s correlation coefficient was calculated. A minimum of 30 cells/sample was analyzed and the statistical analysis was performed using Student’s *t*-test, *** *p* ≤ 0.001. Scale bar, 5 μm. (**B**) Human 2FTGH (2F) fibroblasts, untreated or treated as above, were lysed in lysis buffer, followed by immunoprecipitation with rabbit anti-AMBRA1. A rabbit IgG isotypic control (IpCtr) was employed. The immunoprecipitates were subjected to phospholipid extraction and analyzed by HPTLC immunostaining for the presence of CL. A positive control was obtained using pure standard CL (STD, Sigma-Aldrich). (**C**) The immunoprecipitates were checked for the presence of BECN1 and WIPI1 by Western blot analysis, using anti-BECN1, and anti-WIPI1 Mab. As a control, the immunoprecipitates were assessed by immunoblot with anti-AMBRA1 Mab. A representative experiment among 3 is shown. Bar graph on the right shows densitometric analysis. Results represent the mean ± SD from 3 independent experiments. *** *p* ≤ 0.001, HBSS vs control cells.

In order to clarify the possible role of CL in autophagosome formation, we investigated its association with AMBRA1 in fibroblast cells during the autophagy process. Previously, we had showed that AMBRA1, a key molecule involved in the early events of autophagic flux, is strictly associated with lipid rafts at the MAMs mostly during HBSS-induced autophagy [37]. Thus, in this study, we preliminary investigated the association of CL with AMBRA1 by scanning confocal microscopy analysis. As expected, in untreated fibroblast cells AMBRA1 staining was predominantly diffused in the cytoplasm, while cardiolipin staining was irregular and dotted on the mitochondrial membrane (Figure 3A). However, we observed a weak association of CL with AMBRA1 at preferential mitochondria sites (arrows in merge micrograph, upper panel Figure 3A) in accordance with literature data regarding the existence of a pool of AMBRA1, which was also detected at ER as well as at mitochondria [44,45]. In particular, we observed that the colocalization of CL with AMBRA1 was significantly increased after 1 h HBSS treatment, as revealed by overlapping yellow areas resulting from green and red fluorescence in merge micrograph (arrows in lower panel, Figure 3A). Next, these findings were supported by co-immunoprecipitation experiments. Cells, untreated or treated with HBSS for 1 h, were lysed and subsequently AMBRA1 was immunoprecipitated. The immunoprecipitates were subjected to phospholipid extraction and analyzed by HPTLC immunostaining. The analysis showed in AMBRA1 immunoprecipitates of control cells a positive band of CL coimmunoprecipitation, which was more evident after HBSS treatment, as revealed by densitometric analysis (Figure 3B). No bands were detected in control immunoprecipitation experiments, with IgG having an irrelevant specificity. Immunoprecipitation was checked for the presence of AMBRA1, BECN1 and WIPI1 by Western blot (Figure 3C).

### 3.4. Cardiolipin Associates with MAM Components during Autophagosome Formation in Neuronal Cells

**Figure 4:**
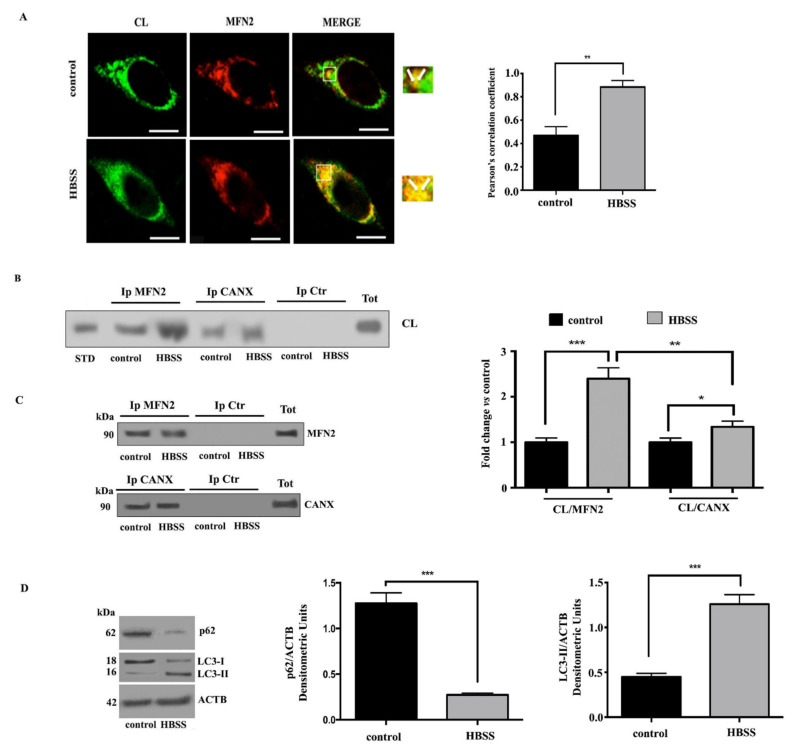
CL interaction with MFN2 and CANX during autophagy induction in neuronal cells. (**A**) SKNB-E-2 cells, untreated or treated with HBSS for 1 h, after fixation with 4% paraformaldehyde in PBS for 30 min at room temperature and then permeabilization by 0.5% Triton X-100 in PBS for 5 min at room temperature, were double stained with human anti-CL and rabbit anti-MFN2 primary antibodies for 1 h, followed by addition of Texas Red-conjugated anti-rabbit IgG and FITC-conjugated anti-human IgG (Sigma-Aldrich) for an additional 45 min at 37 °C. The images were acquired with Olympus FV1000 spectral confocal laser scanning microscope. Pearson’s correlation coefficient was calculated. ** *p* ≤ 0.01. Scale bar, 5 μm. (**B**) SKNB-E-2 cells, untreated or treated with HBSS for 1 h, were lysed in lysis buffer, followed by immunoprecipitation with rabbit anti-MFN2 or rabbit anti-CANX PAb. A rabbit IgG isotypic control (IpCtr) was employed. The immunoprecipitates were subjected to phospholipid extraction and analyzed by HPTLC immunostaining for the presence of CL. A positive control was obtained using pure standard CL (STD, Sigma-Aldrich). (**C**) The immunoprecipitates were checked by Western blot analysis, using mouse anti-MFN2 MAb or mouse anti-CANX MAb. A representative experiment among 3 is shown. Bar graph on the right shows densitometric analysis. Results represent the mean ± SD from 3 independent experiments. * *p* ≤ 0.05, ** *p* ≤ 0.005, *** *p* ≤ 0.001 HBSS vs. control cells. (**D**) Autophagy was checked by Western blot analysis, using rabbit anti-LC3 PAb or rabbit anti-p62/SQSTM1 PAb. Loading control was evaluated using anti-ACTB MAb. A representative experiment among 3 is shown. Bar graph on the right shows densitometric analysis. Results represent the mean ± SD from 3 independent experiments. *** *p* ≤ 0.001, HBSS vs. control cells.

We investigated a possible molecular association of CL with key components of MAMs during autophagy in neuronal cells. With this aim, we preliminary analyzed CL/MFN2 association in SKNB-E-2 cells by scanning confocal microscopy analysis. Likely results obtained above on fibroblast cells, laser confocal microscopy showed an evident association of CL with MFN2 in SKNB-E-2 control cells, as revealed by overlapping yellow areas (see arrows in merge micrograph upper panel, Figure 4A). These areas of colocalization were significantly increased in cells stimulated with HBSS for 1 h (see arrows in merge micrograph lower panel, Figure 4A). 

The association of CL/MFN2 was also confirmed by coimmunoprecipitation experiments. SKNB-E-2 cells, untreated or treated with HBSS for 1 h, were lysed and subsequently MFN2 and CANX were immunoprecipitated. Next, all samples were subjected to phospholipid extraction. HPTLC immunostaining analysis revealed an interaction of CL with both MAMs markers, which was more evident after HBSS stimulus, as also revealed by densitometric analysis (Figure 4B). Immunoprecipitation was checked by Western blot and no bands were detected in control immunoprecipitation experiments with an IgG having irrelevant specificity from both untreated and HBSS-treated cells (Figure 4C). Autophagy induction was checked in control and HBSS-treated cells by Western blot analysis, using anti-LC3 or anti-p62/SQSTM1 antibodies (Figure 4D).

**Figure 5:**
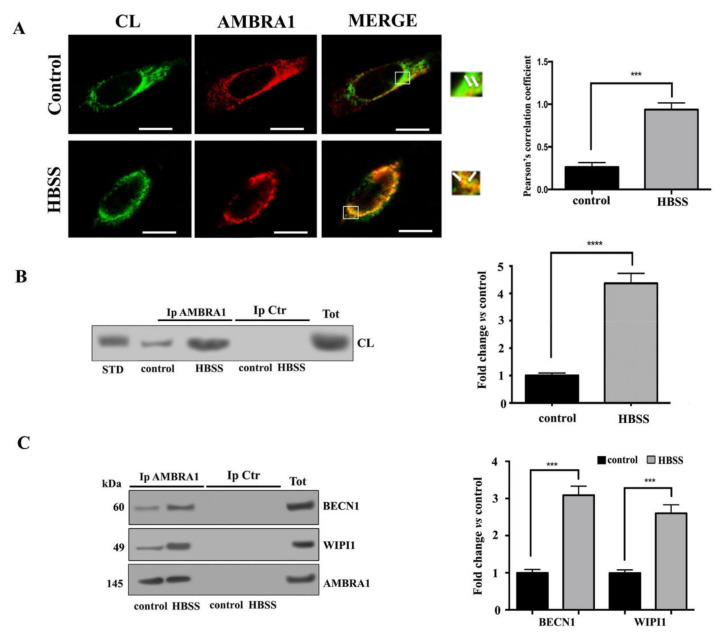
Cardiolipin/AMBRA1 association during autophagy induction in neuronal cells. (**A**) Analysis of CL/AMBRA1 co-localization during autophagy induction in SKNB-E-2 cells, either untreated or treated with HBSS 1 h, after double staining with anti-CL (green) and anti-AMBRA1 (red fluorescence). The images were acquired with Olympus FV1000 spectral confocal laser scanning microscope. Pearson’s correlation coefficient was calculated. *** *p* ≤ 0.001, HBSS vs. control cells. Scale bar, 5 μm. (**B**) SKNB-E-2 cells, untreated or treated with HBSS for 1 h, were lysed in lysis buffer, followed by immunoprecipitation with rabbit anti-AMBRA1. A rabbit IgG isotypic control (IpCtr) was employed. The immunoprecipitates were subjected to phospholipid extraction and analyzed by HPTLC immunostaining for the presence of CL. A positive control was obtained using pure standard CL (STD, Sigma-Aldrich). (**C**) The immunoprecipitates were checked for the presence of BECN1, WIPI1 and AMBRA1 by Western blot analysis, using anti-*BECN1, anti-*WIPI1 Mab and anti-AMBRA1 Mab. A representative experiment among 3 is shown. Bar graph on the right shows densitometric analysis. Results represent the mean ± SD from 3 independent experiments. **** *p* ≤ 0.0001, HBSS vs. control cells.

Next, we analyzed a possible molecular association between CL and AMBRA1 in SKNB-E-2 cells upon autophagy induction. Again, we preliminary analyzed this association by scanning confocal microscopy, which revealed yellow colocalization areas in control cells, which were increased after 1 h HBSS treatment (Figure 5A). Then, SKNB-E-2 cells in fed and starved conditions were subjected to immunoprecipitation using an anti-AMBRA1 antibody. AMBRA1 immunoprecipitates were subjected to phospholipid extraction and analyzed by HPTLC immunostaining. This analysis indicated that the association of AMBRA1 and CL was already present in control SKNB-E-2 cells and increased significantly after incubation with HBSS medium, as revealed by densitometric analysis (Figure 5B). Immunoprecipitation was checked for the presence of AMBRA1, BECN1 and WIPI1 by Western blot and no bands were detected in control immunoprecipitation experiments with an IgG having irrelevant specificity from both untreated and HBSS-treated cells (Figure 5C).

## 4. Discussion

In previous studies we demonstrated that AMBRA1 is recruited to the BECN1 complex and relocalizes to MAMs, where it regulates autophagy by interacting with raft-like components [37,41]. In particular, we showed that the stimulation of autophagy by nutrient starvation leads to relocalization of AMBRA1 at the MAM level together with the BECN1 complex. On MAM, AMBRA1 interacts with lipid raft-like components, such as GD3 and WIPI1, and positively regulates autophagy mainly during the early events. These results confirmed the role of AMBRA1 in the organelle membrane scrambling activity, which finally leads to the formation of autophagosome [37]. The present study adds a new task in this mechanism, by demonstrating that CL is also present in MAM fractions following autophagy triggering and interacts with key molecules involved in autophagosome formation. The presence of CL in the MAM fraction is not surprising, since it is present in the contact sites formed between the inner and outer membranes [6]. The presence of CL in MAM is in agreement with Li et al. [46], who reported that MAM is a major site for phospholipid synthesis and traffic, including CL. This finding highlights a possible implication of raft-like microdomains enriched in CL as a signaling platform in autophagosome formation. Interestingly, we found a molecular association of CL not only with MFN2 and CANX, but also with a AMBRA1/BECN1/WIPI1 multimolecular complex, a crucial regulator within MAM lipid microdomains during the early phases of the autophagic process. CL–protein interaction is a two-step process where electrostatic attraction initiates the interaction of CL with protein, and hydrophobic contacts further consolidate the binding to be of high affinity. In particular it was already shown that BECN1 preferentially associates with CL-enriched membrane [47]. CL-AMBRA1 molecular interaction may be attributable to electrostatic interaction between positive charged amino acids of AMBRA1 and negatively charged CL. 

Emerging evidences indicated that CL may play a role in the pathogenesis of neurodegenerative disease by interaction with specific proteins [48] and that the dysfunction of mitophagy is often associated with neurodegenerative diseases [49,50,51]. In light of this, we translated our study on a neuronal model. Following mitochondrial injury, a significant portion of CL translocates to the outer surface of the mitochondrial membrane, where it interacts with VDAC, Beclin1, and with LC3, one of the autophagy proteins present on the phagophore, thereby mediating the selective enrichment of damaged mitochondria within the forming autophagosome. This condition promotes the degradation of damaged mitochondria through mitophagy, a selective form of autophagy necessary for neuron health, which if left unchecked, can prove harmful by removing too many mitochondria. Although the upstream mechanisms triggering CL externalization in response to HBSS remain to be elucidated, many studies observed that nonoxidized CL is enough to trigger mitophagy [49].

In order to clarify a possible involvement of CL in autophagic machinery within ER-mitochondria tethering we investigated the interaction of CL with MFN2, CANX and AMBRA1. Our results confirmed a significant interaction between CL and MAMs markers, mainly after HBSS stimulus.

This result may be relevant, because CL/AMBRA1 interaction not only occurs at the early phase of mitophagy within MAMs, but this association can also play a role in the late phase, since, upon mitophagy induction, AMBRA1, like CL, binds to the autophagosome adapter LC3 through a LIR (LC3 interacting region) motif to eliminate damaged mitochondria in neuronal cells. 

## 5. Conclusions

Overall, CL association with MAMs may have both structural and functional implications. Indeed, structurally, CL is characterized by two negative charges associated with its 2 phosphatidic acid residues linked by a glycerol bridge and 4 associated fatty acyl chains, which differ in length and saturation. Alteration of CL in mitochondria is associated with a significant decrease of membrane potential and pleiotropic defects in mitochondrial function, including mitochondrial enzyme activities, oxidative phosphorylation, protein import, mitochondrial biogenesis, membrane morphology and dynamics [52]. Functionally, a large amount of evidence has highlighted the transient binding of a-synuclein to cardiolipin. In particular, a-Syn was shown to accumulate at CL-enriched microdomains of mitochondrial membranes and impact mitochondrial function [48]. These new findings demonstrate the presence of CL in the multimolecular complex, enabling autophagosome nucleation at the ER-mitochondria membrane contact sites and introducing a new task to better understand the pathogenesis of neurodegenerative diseases and the possible usefulness of pharmacological interventions to preserve/reconstitute CL content and localization in the disease process. 

## Data Availability

Not applicable.
